# Oxidative Stress—Part of the Solution or Part of the Problem in the Hypoxic Environment of a Brain Tumor

**DOI:** 10.3390/antiox9080747

**Published:** 2020-08-14

**Authors:** Kamil Krawczynski, Jakub Godlewski, Agnieszka Bronisz

**Affiliations:** 1 Department of Neurochemistry, Mossakowski Medical Research Centre, Polish Academy of Sciences, 02-106 Warsaw, Poland; kkrawczynski@imdik.pan.pl (K.K.); jgodlewski@bwh.harvard.edu (J.G.); 2 Department of Neurosurgery, Harvey Cushing Neuro-Oncology Laboratories, Brigham and Women’s Hospital, Harvard Medical School, Boston, MA 02115, USA

**Keywords:** glioblastoma, hypoxia, oxidative stress, cancer niche, tumor microenvironment

## Abstract

Rapid growth of brain tumors such as glioblastoma often results in oxygen deprivation and the emergence of hypoxic zones. In consequence, the enrichment of reactive oxygen species occurs, harming nonmalignant cells and leading them toward apoptotic cell death. However, cancer cells survive such exposure and thrive in a hypoxic environment. As the mechanisms responsible for such starkly different outcomes are not sufficiently explained, we aimed to explore what transcriptome rearrangements are used by glioblastoma cells in hypoxic areas. Using metadata analysis of transcriptome in different subregions of the glioblastoma retrieved from the Ivy Glioblastoma Atlas Project, we created the reactive oxygen species-dependent map of the transcriptome. This map was then used for the analysis of differential gene expression in the histologically determined cellular tumors and hypoxic zones. The gene ontology analysis cross-referenced with the clinical data from The Cancer Genome Atlas revealed that the metabolic shift is one of the major prosurvival strategies applied by cancer cells to overcome hypoxia-related cytotoxicity.

## 1. Introduction

Oxygen levels affect various functions and processes, including cell proliferation, cell differentiation, angiogenesis, and metabolism [1,2,3]. While physiologically normoxic conditions for adult human tissues range widely, they are considerably lower than atmospheric oxygen tension of 21% (about 160 mmHg). Within the central nervous system, this value depends on the region, oscillating between 0.5% O_2_ in the midbrain to 8% O_2_ in the pia [4]. As a result of an imbalance between oxygen delivery capacity and consumption, referred to as hypoxia, elevated oxidative stress occurs [5,6]. Hypoxia often builds up in brain tumors, such as glioblastoma (GBM), resulting in increased necrotic death and the formation of necrotic zones surrounded by tumor cells [7,8]. This specific niche creates a favorable microenvironment for the existence of self-renewing glioblastoma stem-like cells—GSCs, which are essential for tumor initiation, resistance, and recurrence [7,8]. The emergence of perinecrotic/necrotic zones also propagates the development of chemo- and radio-resistance, correlating with poor survival prognosis [9,10].

GBM is the most common and aggressive brain tumor in adults with a short therapeutic window. The recent transcriptomic study uncovered a complex ecosystem of GBM as histologically and genetically distinct anatomic features [11] with far-reaching clinical implications. Leading edge (LE) and infiltrating tumor (IT) zones form the outermost regions of the tumor with tumor cells dispersed among normal cells pose a surgical challenge. In contrast, coexistence with cells from normal brain tissue provides a unique opportunity as a control to understand the extent of transcriptome rearrangement in the tumor core. A cellular tumor (CT) is the major part of the tumor, with a ratio of tumor cells to normal cells ranging from 100/1 to 500/1 [11]. With increasing cell density in the tumor core, the availability of oxygen decreases, creating a niche for pseudopalisading cells (PC) and GSCs enriched in the perinecrotic zone (PZ). Thus, these cells from regions around the dead or dying tissue with undergoing necrosis (NE) are one of the most aggressive despite oxygen limitations. A study by Beppu et al. [12] showed that in GBM patients, oxygenation is significantly lower in intratumoral tissue (9.2 mmHg or about 1.2% O_2_) than in peritumoral areas (17.9 mmHg or about 2.3% O_2_). Cancer cells that not only survive such exposure to low oxygen, but thrive in a hypoxic environment, promote tumor growth and progression of the disease. But most importantly, such adaptation to low oxygen is a barrier for conventional and immunotherapy [13,14]. Why the hypoxic microenvironment creates specific niches where stem cells prefer to reside is not clear. Several large-scale genomics projects on GBM specimens identified a subset of GSCs within the PZ and hypoxic zone, which are distinguished by the specific expression of *CD133* (Prominin 1) and/or *CD44* (Osteopontin) markers [7,8,15]. GSCs can self-renew, differentiate, and are responsible for tumor initiation, progression, and recurrence post-treatment [7,8,15].

Oxidative stress resulting from disturbed oxygen balance leads to the generation of reactive oxygen species (ROS). At a physiological level, ROS function to modulate signal transduction pathways, and regulate the activity of transcription factors and mitochondrial enzymes [16,17]. On the contrary, oxidative stress can induce ROS-driven damage of proteins, lipids, and DNA, leading to genomic instability. Therefore, cancer cells are under constant pressure to maintain a balance between ROS and oxidative-stress response for their survival [17]. Adaptive cellular response to low oxygen levels in both physiological and pathological conditions engage diverse pathways, with one of the most recognized being the hypoxia-inducible factors (HIFs), and endoplasmic reticulum (ER) stress responses [18,19]. HIF-related mechanisms responding to fluctuations in oxygen tension affect GSCs’ capacity to initiate tumors [20]. While an increased HIF-1α expression was observed in both tumor-derived GSCs and neural progenitor cells derived from normal brains, the former also show increased levels of HIF-2α (EPAS1) [21]. Moreover, HIF2α is expressed by the GSCs at oxygen concentrations close to normal in vivo oxygen levels (2–5%). Therefore, within highly heterogeneous GBM tumors, diverse tumor cells cope with a broad spectrum of oxygen tension in their surroundings [22,23], and due to the expression of both HIFs, create an advantage over normal cells in adjusting to the microenvironment.

Decades of research on brain pathology suggested an inverse correlation between the likelihood of cancer and neurodegenerative diseases [24]. These studies described the difference between nonmalignant glial and neural cells and glioblastoma cancer cells in sensitivity to low oxygen. Still, the mechanism of oxidative stress response in the hypoxic tumor niche has not been sufficiently scrutinized. Studying tumor microenvironmental stress response in vivo meets considerable methodological obstacles, as the analysis of bulk tissue masks intricate details of communication between different cell types [25,26,27,28]. At the same time, single-cell approaches or transfer to in vitro culture dissipate the very stress that was the initial goal of investigation. But in situ analysis of transcriptome in distinct histological areas of GBM allow us to correlate patient survival with the oxidative stress response signatures.

Using metadata analysis of transcriptome in different subregions of the GBM retrieved from the Ivy Glioblastoma Atlas Project (IvyGAP), we created the hypoxia/oxygen stress-dependent guide, providing evidence that strictly regulated and specific changes in gene expression affect diverse molecular pathways, which GBM cells adopted to cope with oxidative stress. Our efforts created a list of genes and pathways that are putative targets for the development of anticancer treatment.

## 2. Materials and Methods

To catalog the molecular function of genes differentially expressed in the hypoxic regions of the tumor, we conducted a systematic review using the Gene Ontology (GO) database (December 2019 edition). This strategy identified gene sets whose function is related to hypoxic and oxidative stress. Genes were included if the standard nomenclature identified them for human genes in the HGNC database. To create the oxidative stress hypoxic map, we used a curated list of 278 genes whose expression could be found in the IvyGAP. The clinical and genomic database IvyGAP [11] was created for the 41-patient cohort with diagnosed GBM. In brief, we used data plotted in the tumor’s anatomic features (based on in situ hybridization (ISH), followed by the analysis of the transcriptome by laser microdissection (LMD) and RNA sequencing (RNA-seq)). The geographic regions within glioblastoma specimens were separated based on several factors, including gene ontology enrichment analysis, gross tissue pathology, and hypoxic marker *EPAS1* and carbonic anhydrase 9 (*CA9*) [11,26,29]. Thus, a curated list of 278 oxidative stress genes was applied to the matrix of anatomic features to read their expression.

**Figure 1:**
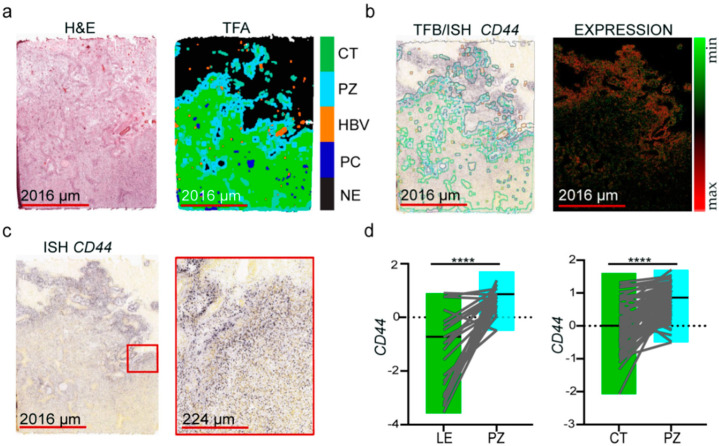
Hypoxia-related gene signature distinguishes between different anatomic features of glioblastoma: (**a**) representative hematoxylin and eosin (H&E) staining section from tumor W5-1-1-L.1.05 (left panel), converted to tumor feature anatomic (TFA) color map (right panel). Color code: green—cellular tumor (CT); light blue—perinecrotic zone (PZ); orange—hyperplastic blood vessels (HBV); purple—infiltrating tumor (IT); dark blue—pseudopalisading cells (PC); black—necrosis (NE); (**b**) representative section labeled using ISH probe against *CD44* gene with merged tumor feature boundary (TFB) of anatomic regions (left panel), and ISH-labeled *CD44* signal converted to fluorescent map (right panel); (**c**) representative section labeled using ISH probe against *CD44* gene (left panel) with red inset indicating a site of magnification; (**d**) RNA-seq expression data of *CD44* (*z*-score values as in Table S1) in anatomic features LE (*n* = 43), CT (*n* = 111), and PZ (*n* = 66) isolated by LMD; paired two-tailed *t*-test, *p*-value between pairs (*n* = 23 for LE vs. PZ, and *n* = 65 for CT vs. PZ) is shown; **** *p* < 0.0001.

The copy number and mutation analyses previously indicated that LE and IT samples mostly consist of non-neoplastic cells. In contrast, CT, PZ, and PC comprise mostly of tumor cells. Due to similar cell composition of some anatomic features of GBM [11], we decided to combine data from LE and IT as well as from PZ and PC, and to consider them as one region. This combination was dubbed LE and PZ, respectively. Thus, all the comparisons were made between LE vs. PZ and CT vs. PZ. Next, we performed differential expression analysis to define gene signatures using cutoff based on a significant difference in the expression of genes from these regions based on *z*-score log-transformed, normalized expression values downloaded from IvyGAP [11]. To set up a hypoxic niche signature into a broader clinical context, we queried the list of genes with The Cancer Genome Atlas (TCGA) data, where the 489 GBM tumors and ten control specimens were analyzed using Affymetrix HT_HG-U133A microarray gene chips. The collection of the data from TCGA [30] and the Ivy Glioblastoma Atlas Project [11] was compliant with all applicable laws, regulations, and policies for the protection of human subjects. GO, and KEGG pathway analyses were performed with the software implemented in ShinyGo v0.61 software. Kaplan–Meier survival analysis was performed using GBM biodiscovery portal [31] for oxidative response genes signature according to Verhaak et al. [30] explained in supplementary materials and methods file. In some cases, genes were absent in the GBM biodiscovery portal dataset; therefore, the final list of genes used for analysis was reduced (*n* = 17 in LE vs. PZ and *n* = 17 in CT vs. PZ comparisons for upregulated genes, and *n* = 19 for CT vs. PZ comparison for downregulated genes). All the statistical operations were performed with the GraphPad Prism 8 software, using paired *t*-test or multiple *t*-test analysis, and considered significant with a *p*-value < 0.05 or False Discovery Rate (FDR) with corrected *q*-value < 0.01, respectively. The in situ hybridization *CD44* and *HIF-1α* signals converted to fluorescence (Figure 1b and Figure S1b, right panels, respectively) were subjected to graphical enhancement (contrast and brightness correction).

## 3. Results

### 3.1. Molecular Markers of GSCs Are Associated with the Hypoxic Zone of Glioblastoma

Hypoxia is a common feature of solid tumors [32]; therefore, we claim that distinct GBM areas affected by diverse oxygen levels could serve as a good model for studying cellular adaptation to oxidative stress. HIF-1α is known as a master regulator of hypoxia [33], whose level/activity was also shown to be upregulated in GBM, particularly around necrotic regions [34]. Therefore, we compared the *HIF-1α* gene expression between LE vs. PZ and CT vs. PZ areas (Figure S1). However, neither in situ hybridization (Figure S1b,c) nor transcript expression (Figure S1d) showed consistent upregulation in PZ. The likely level of stabilized protein could be more relevant [18,35]; however, no data was present in searched databases. Therefore, in the search for other suitable markers of hypoxia in GBM, we compared the expression of *CA9* (Figure S1e) and GSC marker *CD44* [15] in the same anatomic features, as it was proposed in the human and a mouse model of GBM that perivascular and perinecrotic zones are GSC-enriched [15,36,37]. The representative tumor section with H&E staining for the GBM regions is presented in Figure 1a. Both ISH and RNA-Seq data (Figure 1b–d) demonstrated that diverse regions of the tumor can be delineated based on *CD44* and *CA9* expression whose pattern was correlated (Figure S1e). The highest expression of *CD44* found in the PZ (Figure 1d) is consistent with the prevalence of GSCs in this area; thus, it is regarded as a marker for hypoxia as suggested elsewhere [36].

### 3.2. The Expression of Oxidative Stress-Related Genes in GBM Depends on the Microenvironmental Niche

**Figure 2:**
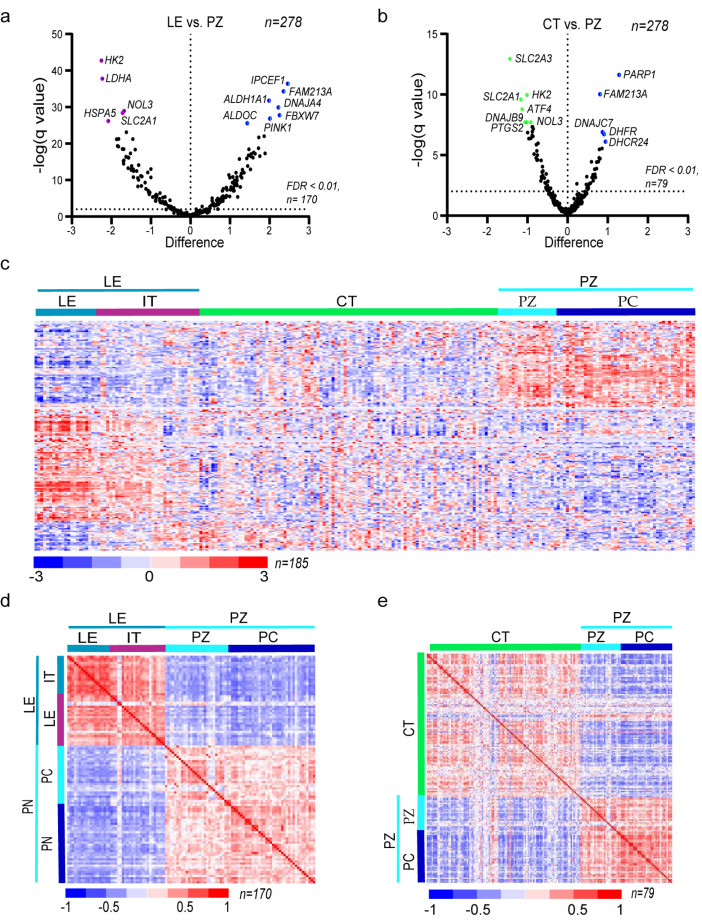
Oxidative stress-related gene expression patterns differ between anatomic features of glioblastoma: (**a**,**b**) volcano plots representing differentially expressed genes between LE vs. PZ (pink—genes downregulated, blue—upregulated in LE) (**a**), and CT vs. PZ (green—genes downregulated and, blue—upregulated in CT) (**b**)*,* each dot corresponds to one gene (*n* = 278), the horizontal dotted line shows a cutoff for FDR < 0.01; (**c**) hierarchical clustering of 185 genes found as statistically differentially expressed between LE, CT vs. PZ in all anatomic features of glioblastoma; rows and columns represent genes and samples, respectively. Colors correspond to the expression level of detected genes, and the blue–red scale bar indicates expression level, with blue being the lowest and red the highest; (**d**,**e**) a correlation matrix showing correlation coefficients between gene expression in LE vs. PZ (*n* = 170; Pearson r = −0.407) (**d**), and CT vs. PZ (*n* = 79; Pearson r = −0.09) (**e**); blue–red bar scale indicates correlation coefficients level, with blue being the negative and red positive correlation.

To better understand cancer cell adaptation to oxygen deprivation, we analyzed expression profiles of genes associated with oxidative stress response in distinct GBM anatomic features. Using a curated list of 278 oxidative stress response genes (Table S1), we performed a cluster analysis based on expression data from RNA-Seq deposited in IvyGAP. Three significant clusters of oxidative stress response genes were evident within the signature discriminating between LE, CT, and PZ (Figure S2a), thus providing the rationale for comparison of the PZ signature with both LE and CT. Following that, differential analysis between subareas of the tumor (Figure 2a,b; Table S2) allowed selection of oxidative stress response gene profiles in different areas of the tumor (Figure 2c). The number of differentially expressed genes, and differences in the magnitude of expression, were more prominent between the PZ zone enriched in GSCs and the LE zone enriched in normal brain cells (*n* = 170, Figure 2d). Such a signature is thus associated with cell-type-dependent oxidative stress response genes profile. While the comparison of CT and PZ provided the signature of genes (79 genes, Figure 2e) that are used by cancer cells in response to oxidative stress in hypoxia. Changes in the GBM transcriptome create specific patterns that mirror the adaptation of cells to the different oxygen gradients in studied regions and can serve as potential markers or therapeutic targets. The observed changes in the GBM oxidative stress response transcriptome created specific patterns that reflect the adaptation of cells from tumor microenvironment to the different oxygen gradients.

### 3.3. Oxidative Stress-Related Genes Signature Predicts the Outcome of GBM Patients

**Figure 3:**
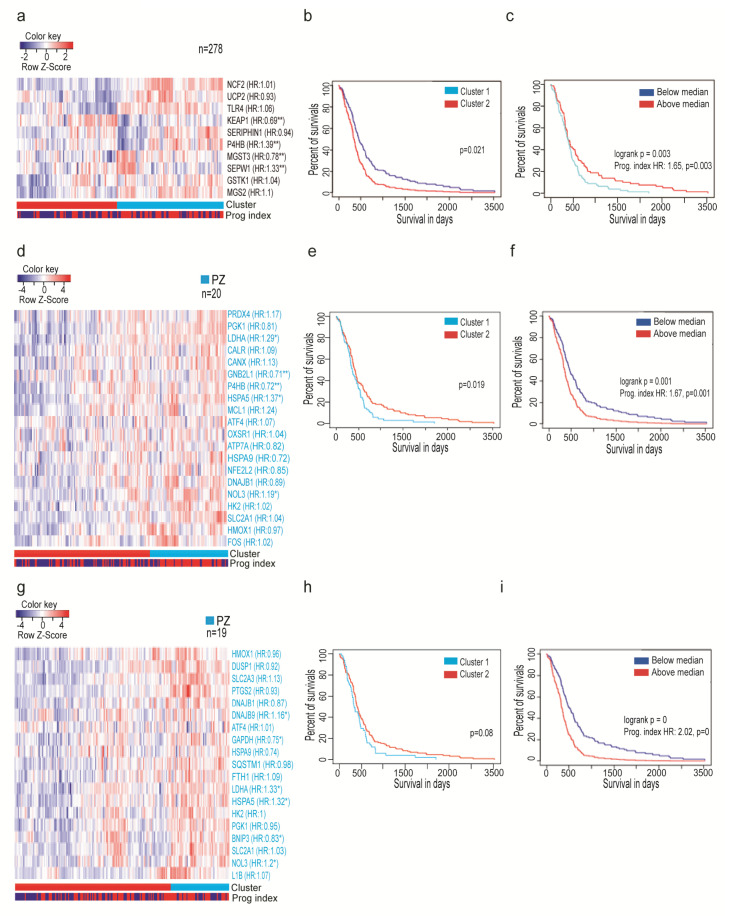
Patient survival is correlated with the expression of oxidative-stress-related genes in the perinecrotic zone: (**a**,**d**,**g**) Heatmaps with color annotations according to profile similarity (light blue/red) annotated with prognostic index (red–dark blue). (**b**,**c**,**e**,**f**,**h**,**i**) Kaplan–Meier analysis of a full cohort of glioblastoma. Clustering and survival analysis of samples dataset stratified by the status of oxidative stress response genes (**a**–**c**), top-20 upregulated genes in PZ vs. LE (**d**–**f**), or vs. CT (**g**–**i**).

To verify whether differential expression of oxidative stress genes has clinical implications, we associated signature with survival outcomes for patients with GBM. We used an admixture model of either oxidative stress gene signature (Table S1), or top-20 genes deregulated in the PZ zone (Table S2) queried with TCGA GBM. The first strategy allowed stratifying patients according to profile similarity and prognostic index based on the most different molecules amongst the samples (Figure 3a). A list of 278 genes was filtered to keep the most varied molecules amongst the samples. Following that, survival analysis based on cluster membership (Figure 3b) and the impact of the multigene prognostic index (Figure 3c) showed the power of outcome prediction of ten-gene oxidative stress signature. The second strategy was performed to find whether the loss or gain of oxidative stress genes signature in the hypoxic region has the power of prediction for patient outcome. This analysis revealed significant power of genes upregulated in the PZ zone in comparison to the LE zone to predict patient outcomes (Figure 3d–f) and to a lesser extent for genes upregulated in PZ compared to CT (Figure 3g–i). In contrast, genes up- and downregulated taken together (Figure S3a–f), or only downregulated in the PZ region, had no such power (Figure S3g–l). These analyses underlined that response to oxidative stress in hypoxic tumors could be a potential source of biomarkers and therapeutic targets. While such multigene analysis identified a group of genes with prognostic value (Figure 3 a–f), only 20 out of 29 genes analyzed separately showed any prognostic value (Table S3). Several genes, whose levels were increased in PZ such as Lactate Dehydrogenase A (*LDHA*), Calnexin (*CANX*), Nucleolar Protein 3 (*NOL3*), Heat Shock Protein Family A (*Hsp70*) Member 5 (*HSPA5*) (Table S2), were also upregulated in patients with poorer prognosis (Table S3). Despite a significant difference in the expression of, e.g., Aldehyde Dehydrogenase 1 Family Member A1 (*ALDH1A1*), *F-Box*, and WD Repeat Domain Containing 7 (*FBXW7*), between LE and CT vs. PZ (Table S2), none of these genes correlated with the survival of GBM patients (Table S3). It suggests that the adaptation to oxidative stress to overcome hypoxia is achieved by the active upregulation of a distinct set of genes that creates microenvironment favorable for tumor progression.

### 3.4. Oxidative Stress Response Genes Have Different Functions in Diverse Microenvironmental Niches

**Figure 4:**
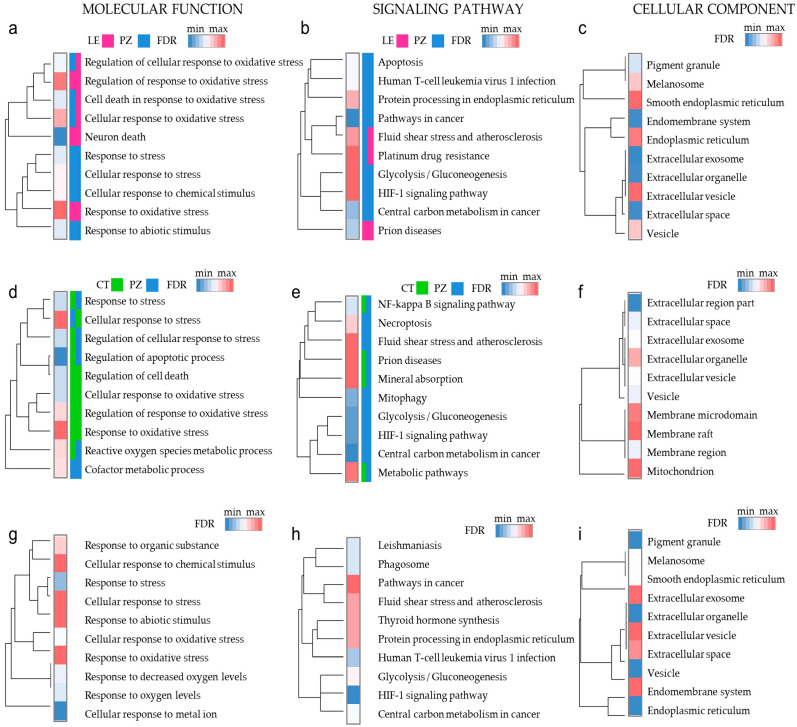
Gene Ontology analysis reveals activation of distinct pathways in diverse anatomic features of glioblastoma: Function of top-20 (**a**–**f**) and 29 outcome predictive gens (**g**–**i**) represented as Gene Ontology tree in indicated categories. Color bars (**a**,**b**,**d**,**e**) represent an enrichment of upregulated genes from different anatomic tumor features (LE-pink, CT-green, PZ-blue), a single-color square indicates activation of unique pathways, and a two-color square indicates shared pathways. FDR values are shown as a heat map, with red maxim and blue minimum.

The oxidative stress response is a network process employing apoptosis and autophagy pathways and metabolism rearrangements. To answer what mechanisms are used by cancer cells to thrive in a hypoxic niche, we scrutinized the ontology of genes associated with different areas of the tumor. As our list of genes was already preselected for those enriched in the GO terms related to hypoxia and oxidative stress, we first narrowed our analysis to the top-20 model (Table S2 (LE vs. PZ and CT vs. PZ)). The gene ontology analysis visualizes the connection between studied genes in Figure 4a–f and Figure S4a,b. “Metabolism” and “oxidative stress response” were found to be among the most enriched categories (Figure 4a,b,d,e, Table S4). Genes whose expression was upregulated in PZ (blue color in bar legend) were included in processes termed “neuron/cell death” and “metabolism” such as necroptosis, mitophagy, glycolysis/gluconeogenesis, and carbon metabolism. Besides, as visualized by Venn diagrams (Figure S4c; Table S5), many of these genes were commonly affected in both LE and CT. The second analysis was performed to find out whether clinically relevant oxidative stress genes signature have the potential to bring us closer to pinpointing therapeutic obstacles (Table S3, Figure 4g,h). Interestingly, proteins encoded by genes with prognostic value and most deregulated genes in LE, CT vs. PZ (Figure 4c,f,i) show functional enrichment as bridging molecules secreted into extracellular vesicles. That is important as vesicles transferred over a considerable distance may propagate hypoxic niche signaling, so by targeting PZ-specific genes, we can expect a therapeutic benefit in CT tumor.

## 4. Discussion

Our study linked the oxidative-gene expression signature altered in the PZ zone of glioblastoma with complex crosstalk between key signaling pathways, which in turn may promote tumor survival in the hostile microenvironment. These processes include a shift in the metabolism towards glycolysis [38,39], induction of autophagy, and protection from apoptotic cell death [40,41] that all have been proposed to be of particular importance in cancer. By exploring transcriptome rearrangements in different anatomic regions of GBM, we showed what genes may predispose tumor cells to survive in hypoxic conditions under oxidative stress.

As GBM is a highly heterogeneous tumor and diverse cells across the tumor cope with a full spectrum of oxygen tension in their surroundings [22,23], we first sought for a relevant marker of hypoxia, whose expression could distinguish between LE, CT, and PZ. We showed that high levels of *CD44,* a well-known GSC marker [6,7], were prevalent in PZ, in line with the notion that GSCs are enriched within a hypoxic tumor niche where the oxidative stress is elevated [7,8]. In contrast, *HIF1α* expression was inconsistent in studied regions. Yet, *HIF1α* and *HIF2α* are well-known master regulators of hypoxia, and once induced under hypoxic conditions [42], they drive expression of multiple hypoxia-responsive genes [21,42].

As evident from our analysis of differential gene expression and GO and KEGG pathways, the adaptation of GBM cells to low oxygen microenvironment could be achieved by enhanced and modified metabolism, as well as by maintaining the equilibrium between activation and inhibition of stress response pathways. Interestingly, as few as 20 most upregulated genes in PZ was sufficient to predict outcome in GBM patients. It suggests that the upregulation of a distinct set of genes in the hypoxic tumor core orchestrates its adaptation to oxidative stress and creates a specific microenvironment for tumor development.

It was proposed almost a century ago that tumor cells may adapt to the unfavorable low oxygen environment by switching their metabolism between mitochondrial oxidative phosphorylation and aerobic glycolysis (“the Warburg effect”) [38]. Activation of HIFs leads to the expression of glycolytic factors, including glucose transporter-1,3 (*Glut-1,3* aka *Slc2a1, 3*); aldolase C (*ALDOC*); glyceraldehyde phosphate dehydrogenase (*GAPDH*); hexokinase 1,2 (*HK1,2*); lactate dehydrogenase-A (*LDHA*); phosphofructokinase L (*PFKL*); and phosphoglycerate kinase 1 (*PGK1*) [43,44]. Overexpression of these genes in the PZ area evident from our analysis, strengthen the notion that specific expression patterns of those genes may promote cancer survival in the hostile microenvironment. For instance, LDHA is an enzyme responsible for the conversion of pyruvate to lactate under anaerobic conditions and is key in the altered glycolytic metabolism. The silencing of LDHA expression in GBM resulted in reduced glycolysis, decreased cell growth, and increased cell apoptosis [45,46,47]. A complementary action to maintain balanced glucose levels may require the downregulation of some other genes. Per our analysis, FBXW7 was one of the most affected genes in the PZ area of GBM. The knockdown of FBXW7 promotes malignant phenotypes in vitro and in vivo, e.g., by indirect targeting of c-MYC, leading to reduced glucose uptake [48].

Nonetheless, such dynamic and rapid changes in metabolism often lead to the accumulation of toxic byproducts. We found that ALDH1A1, belonging to the superfamily of enzymes responsible for the catalysis of aldehyde oxidation [49], whose accumulation can be toxic to cells, was significantly downregulated in the PZ area. ALDH1A1 specifically catalyzes the oxidation of retinaldehyde to retinoic acid, and the latter was shown as an effective treatment against GBM cells by inducing their differentiation, reducing the expression of stem cell markers like CD133, CD44, and Sox-2, and decreasing the neurosphere-forming capacity [50]. Therefore, yet another metabolic adaptation applied by cancer cells is the reduced rate of synthesis of such metabolites by decreased expression of the ALDH1A1 gene.

Several approaches have been applied to target metabolism in glioblastoma as well as other cancers [51,52,53]. Different studies showed that deletion/inhibition of LDHA combined with treatment with drugs such as tamoxifen, taxol, or use of drugs alone (oxamate, phenformin, gossypol, galloflavin) could impact tumorigenesis by affecting glucose uptake and increasing tumor apoptosis (reviewed in [51,52,53]). Gossypol, for instance, is well tolerated in clinical trials and has shown promise in recurrent malignant glioma trials [54]. Combinations of rapamycin with chemotherapy (temozolomide, doxycycline, etomoxir) were shown to be effective strategies in GBM [53]. However, the common problem with drugs is that they have limited cell penetration; therefore, relatively high doses are required to have any significant effect. Nonetheless, enhanced search for alternative drugs, both of natural origin [55,56] or less toxic/more potent derivatives may provide a successful therapy for glioblastoma. The link between oxidative stress and inflammation has been described in the literature in various cancers [57,58]. The regulation of expression of proinflammatory cytokines is a consequence of crosstalk between HIF-related transcriptional activity, and the transcription factor NF-κB (nuclear factor-kappa B) pathway [59]. Our differential expression analysis showed significant upregulation of immune-response-related factors, which indicate possible crosstalk between those pathways in GBM. For instance, proinflammatory PTGS2 (better known as COX2) is an enzyme engaged in the biosynthesis of prostaglandin E2 (PGE2), which plays a vital role in modulating of motility, proliferation, and resistance to apoptosis [60,61]. It has been shown that in GBM, PTGS2/COX-2-positive cells accumulated in perinecrotic regions of the tumor [62]. High levels of PTGS2 in GBMs are positively correlated with many aggressive traits of the disease, such as the cell proliferation rate or GBM grade [60,62]. Both chemotherapy and radiotherapy induce COX-2 to synthesize PGE2 in GBM cells to produce immunosuppressive cytokines, such as interleukin 6 (IL-6) and IL-10, which are upregulated in the PZ region per our analysis, blocking T cell functions [62]. These findings might be of particular interest as GBM is considered to be one of the most immunosuppressive tumors, characterized by reduced infiltration and proliferation of immune cells [63,64]. Some of the current approaches include stimulation of the immune system, e.g., by use of immune checkpoint inhibitors. At the same time, other approaches, such as chimeric antigen receptor (CAR) T-cell therapies, involve the use of individually engineered T-cells to attack cancer cells [65,66]. Despite continued research and some promising initial results of novel therapies, none have significantly impacted patient mortality in GBM to date.

Cell proliferation and death are inherent characteristics of all cells, including malignant ones. We found that many genes whose expression was affected per our analysis were related to cell death. Three main categories recognized as programmed cell death include autophagy, apoptosis, and necroptosis, and an imbalance between cell death and survival is a crucial step of cancer initiation [67,68]. Several studies showed that GBM cells were characterized by increased expression of antiapoptotic proteins such as FOS (Fos proto-oncogene), MCL-1 (Myeloid Cell Leukemia 1), or NOL3 (Nucleolar Protein 3). Their expression was shown by our study to be increased in the PZ, and their knock-out sensitized GBM cells to radiation or chemotherapy, induced senescence, and enhanced apoptosis [69,70].

As demonstrated in the cell culture model, factors secreted by GBM cells can induce oxidative stress in surrounding nontumor cells and trigger apoptotic pathways in them [71]. That corresponds with our findings that proapoptotic genes were among mostly upregulated genes in LE or CT, whereas antiapoptotic genes were mostly upregulated in PZ. Our analysis also showed increased expression of several autophagy-related genes in PZ compared to other regions, such as B-Cell CLL/Lymphoma 2 interacting protein (*BNIP3*), Activating Transcription Factor 4 (*ATF4*), Sequestosome 1 (*SQSTM1*), and Autophagy Related 7 (*ATG7*), whose induction acts as a mechanism preventing cell death through apoptosis [40,72,73,74]. Interestingly, there are links between apoptosis and metabolic pathways. For instance, LDHA reduction resulted in an inhibited cancer cell proliferation, elevated cellular oxidative stress, and induction of apoptosis via the mitochondrial pathway [75]. Moreover, it was shown that tumor cells, resistant to apoptosis, undergo necrosis when exposed to excessive metabolic stress [76,77], and that an increase in autophagy suppresses the necrosis [40,41]. Different approaches to augment intrinsic or induced apoptosis has been proposed to apply as a possible therapy against GBM [78,79]. Although apoptosis can be a measure of cell death, it is disputable whether it has a prognostic value for patient outcome in GBM [80,81].

Complex tumor microenvironment and heterogeneity of glioblastoma are critical problems in the design of successful therapy. We thus emphasize that diverse molecular pathways are turned on in different tumor regions/anatomical features; thus, a combination of treatments and approaches rather than single-gene targeting will bring us closer to a breakthrough in the struggle against GBM.

## 5. Conclusions

Through altering the expression of diverse genes, cancer cells activate complex crosstalk that intertwines metabolism and other key signaling pathways. These include cell death, immune response, and protein processing. Therefore, studying the dynamic adaptation of tumor cells to fluctuating microenvironmental factors such as nutrient supply, oxygen level, or treatment-inflicted damage is challenging. A plethora of genes whose expression is changed in the GBM are potential targets for therapy. Proteins encoded by oxidative stress genes with prognostic value identified by our study are secreted via extracellular vesicles into microenvironment [82,83,84,85], implying that targeting of expressed and secreted factors from PZ will bring therapeutic benefit to CT tumors. However, pathways adopted by cancer cells to overcome hypoxic stress are also common among normal cell types, and GBM cells somehow manage to master their regulation. We believe that our analysis and systematic update of the knowledge may help to understand the complex microenvironment of GBM better and create means to develop potential therapeutic strategies to target tumor cells. However, the lingering questions remain: what are the alternative strategies engaged upon the exposure to various intrinsic or extrinsic oxygen/nutrient deprivation? Do different cell populations within the tumor have different strategies to overcome oxidative stress? How do cancer cells maintain a balance between activation and suppression of diverse pathways to support their growth? Can cells modify particular pathways to overcome the exposure to adverse microenvironmental conditions?

Genes listed in this study were analyzed based on RNA expression, not protein levels, which certainly might alter their ranking of importance. The expression of HIF1a transcript is not associated with the prognosis of glioblastoma, but hypoxic cancers have a poorer overall prognosis [86]. This paradigm implies that for protein-coding genes, the protein level and or activity affected by post-translational modifications or copartner binding is a crucial indicator of function. While awaiting a much-anticipated atlas of active protein within glioblastoma features, we can learn more about the intricacies of glioblastoma using more feasible to profile noncoding RNAs [27,83,87].

## Supplementary Materials

The supplementary materials are available online at https://www.mdpi.com/2076-3921/9/8/747/s1.
