# Supplementary material for: Oxidative Stress—Part of the Solution or Part of the Problem in the Hypoxic Environment of a Brain Tumor
Source: Antioxidants (Basel). 2020 Aug 14;9(8):747. doi: 10.3390/antiox9080747 (PMC7464568; doi:10.3390/antiox9080747)
Supplement: Supplementary file 1 [file antioxidants-09-00747-s001.zip › antioxidants_supp_877749-rev 2.docx]

Supplementary data Metadata/Review

Supplementary Materials and Methods and Database Information:

The software used in the study: For all studies except statistical analysis, we used free-access online data resources that served as a platform for our investigation of hypoxia driving pathogenesis and treatment of GBM.

TCGA [[1]]: <https://www.cancer.gov/>

Ivy GAP [[2]]: <http://glioblastoma.alleninstitute.org/rnaseq>

GBM biodiscovery portal [: <https://gbm-biodp.nci.nih.gov>

dchip: <http://www.dchip.org/>

Gene Ontology database: <http://www.geneontology.org/>

GlioVis [[3]]: <http://gliovis.bioinfo.cnio.es/?ref=labworm>

HGNC database: <http://www.genenames.org>

ShinyGO v0.61: <http://bioinformatics.sdstate.edu/go/>

STRING [[4]]: <https://string-db.org/>

Details of Data analysis: The collection of the data from IVY GAP and TCGA GBM was compliant with all applicable laws, regulations, and policies for the protection of human subjects, and necessary ethical approvals were obtained. For analysis of gene expression in glioblastoma, we used normalization of data and aggregation at the feature level as designated by the TCGA GBM the "Level3". Data were analyzed using free available portals as a resource for accessing and displaying interactive views of Ivy GAP and TCGA data associated with glioblastoma [[1,2]]. Z-score log-transformed normalized RNA-Seq expression values from IvyGAP were used for the analysis.

Displaying a summary of experimental data associated with selected genes: The samples (columns on the heatmap) were annotated in two ways: first, according to the cluster membership (the optimal number of clusters was determined using NbClust); second, by inspecting the status of a prognostic index (which was computed by weight averaging the gene expressions with the regression coefficients of a multi-gene Cox proportional hazards model). The gene names were annotated with their respective Hazard Ratios in a multi-gene Cox proportional hazards model.

Performing gene survival analysis: The Kaplan-Meier survival curve analysis compared samples stratified according to gene expression levels. The default options stratified samples into two groups: those with expression levels below the median over the subgroup, and those with expression levels above the median. For gene searches that resulted in multiple hits, we analyzed how the expression profiles impacted the survival. We performed two types of survival analyses: first, the optimum clusters were selected by the stratification of the samples according to the heatmap cluster membership (see the first annotation bar), where the optimal number of clusters is picked out algorithmically using silhouette width index. Next, we used a Kaplan-Meier model to analyze the differences in survival between groups using a log-rank statistic. Hierarchal clustering analysis of a full cohort of IvyGAP samples dataset stratified by inspecting the status of genes with the most varied prognostic index that was computed by weight averaging the gene expressions with the regression coefficients of a multi-gene Cox proportional hazards model.

Displaying heatmap clustering of gene expression data correlation: For selected multiple hits of genes, we presented a heatmap of the correlation between the expression of genes in anatomic features using dChip. Each cell of the heatmap represented how the expression of the gene in the row, and the gene in that column are correlated, and it was annotated with the correlation value. The results were displayed as a heat map using hierarchical clustering analysis using the average linkage distance metric.

Displaying gene networks: Quality-controlled protein-protein association networks STRING (Search Tool for the Retrieval of Interacting Genes) [[4]] has been used to visualize the interaction between differentially expressed genes between studied anatomical features of glioblastoma. The minimum required interaction score was set for high confidence (0.7), and k-means clustering of genes was used. Color nodes correspond to query proteins and the first shell of interactors. Edges represent protein-protein (gene-gene) association.

Quantification and Statistical Analysis: Graphs and plots were generated, and statistical analyses were performed using GraphPad Prism 8. Statistical parameters, including the value of n, statistical test, and statistical significance (*p*-value and FDR), are reported in the figures and their legends. Statistical tests were selected based on the desired comparison. Paired two-tailed *t-*tests were used to assess significance when comparing data between two variances. For the differential expression of multiple measurements, the multiple *t-*test was performed using the False Discovery Rate (FDR) approach with a two-stage step-up method of Benjamini, Krieger, and Yekutieli [[5]] to correct for multiple hypothesis testing. Differences were considered significant with *p*-value<0.05 and FDR<0.01, respectively.

Supplementary Figures.


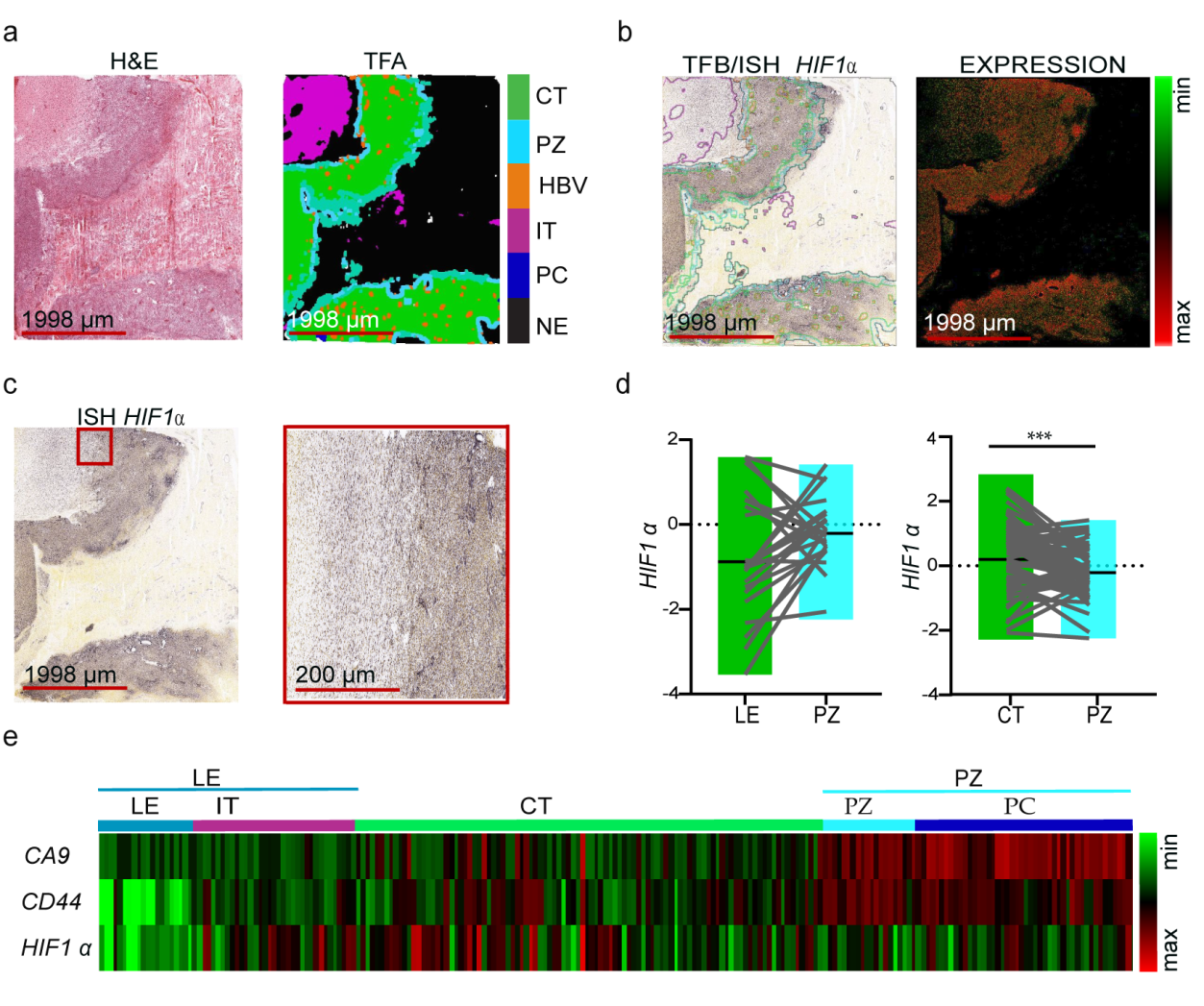


**Figure S1. Supplementary Figure to Figure 1:** **(a)** representative H&E staining section (left panel), converted to TFA color map (right panel); **(b)** representative section labeled using ISH probe against HIF1α gene with merged tumor feature boundary of anatomic regions (left panel); and ISH labeled HIF1α signal converted to fluorescent map (right panel); **(c)** representative section labeled using ISH probe against HIF1α gene (left panel) with red inset indicating site of magnification on the right; **(d)** RNA-seq expression data of HIF1α (z-score values as in Table S1) for LE (n=43), CT (n=111), and PZ (n=66) features isolated by LMD, paired two-tailed *t*-test, *p*-value between pair (n=23 for LE vs. PZ, and n=65 for CT vs. PZ) is shown, *** p=0.0003; **(e)** heat map of three genes used as markers associated with hypoxia in all anatomic features of GBM; scale bar shows a range of the expression represented as *z*-score with green as low and red as high expression.


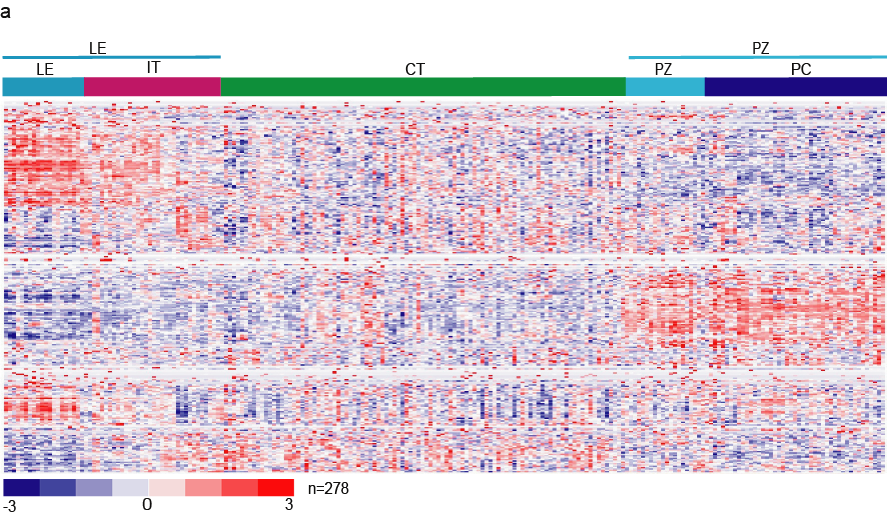
**Figure S2. Supplementary Figure to Figure 2:** **(a)** hierarchical clustering of 278 pre-selected genes associated with oxidative stress in all anatomic features of GBM; scale bar below heatmap shows a range of the expression of studied genes with blue as low and red as high expression.


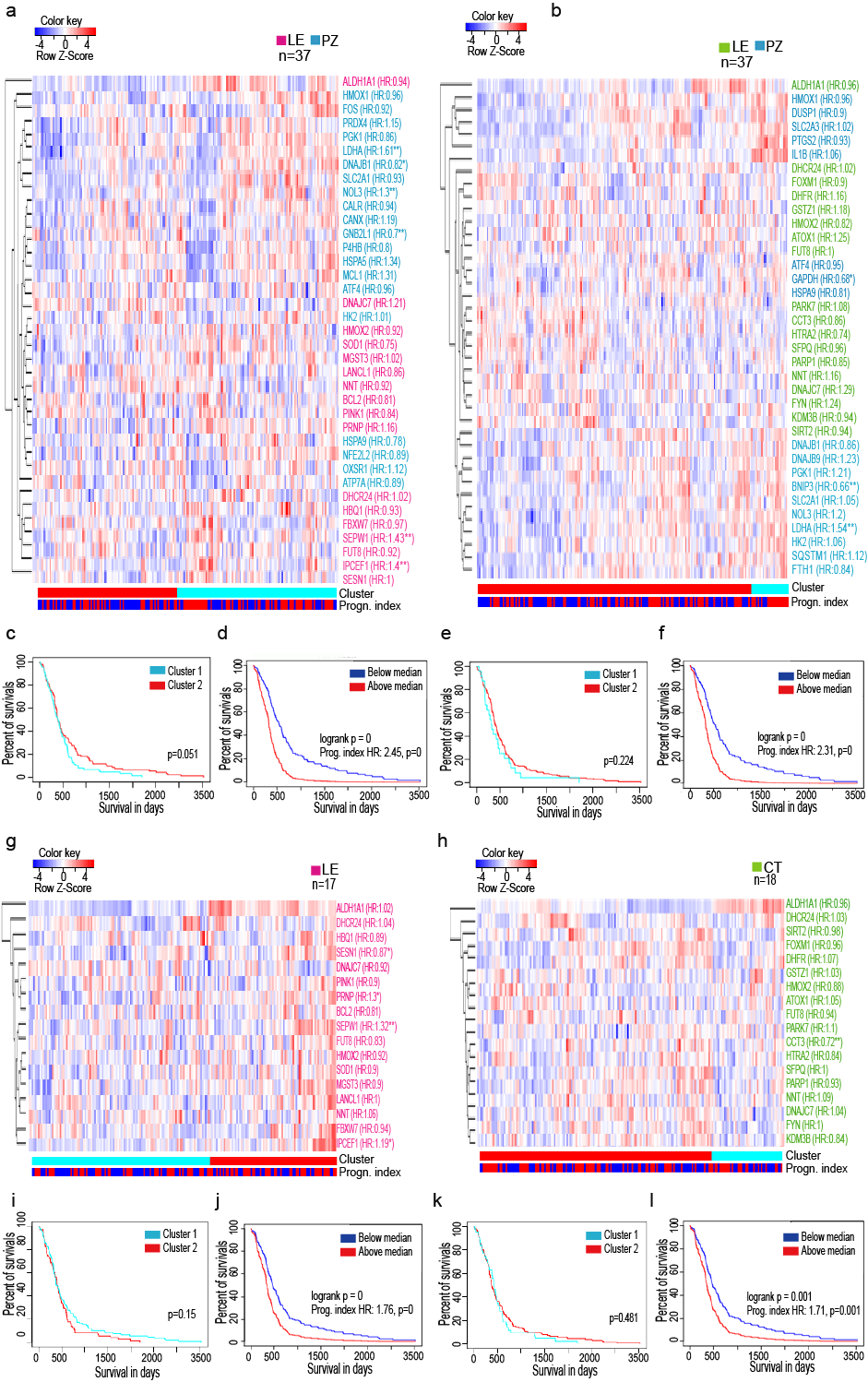


**Figure S3. Supplementary Figure to Figure 3:** (**a, b, c, d**) Heatmaps with color annotations according to profile similarity (light blue/red), and annotated with prognostic index (red-dark blue). (**c, d, e, f, i, j, k, l**) Kaplan-Meier analysis of a full cohort of glioblastoma. Clustering and survival analysis of samples dataset stratified by inspecting the status of differentially expressed oxidative stress response genes (**a, b, c, d, e, f**) and top-20 downregulated genes in PZ *vs.* LE (**g, i, j**) or *vs.* CT (**h, k, l**).

|  |  |
| --- | --- |


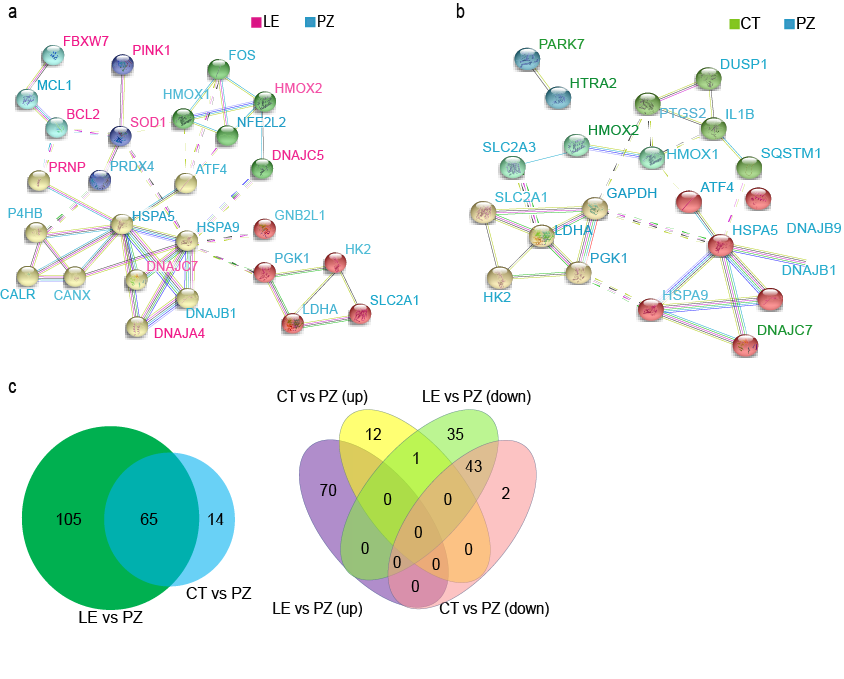
**Figure S4. Supplementary Figure to Figure 4:** **(a, b)** Association of top-20 deregulated genes represented as STRING gene network, **(c)** Venn diagrams representing differentially expressed genes between LE *vs*. PZ and CT *vs*. PZ. Green and blue circles (left) represents all deregulated genes in indicated comparisons, and ovals with different colors (right) represent either up- or down-regulated genes in studied comparisons.

Supplementary Tables

**Table S1. A curated list of genes from the Ivy Glioblastoma Atlas Project with gene expression.** A list containing gene expression values represented as *z*-score [[1]]. Rows display gene symbols and official names. Columns correspond to patient-derived samples. LE, IT, PZ, and PC, indicate an anatomical feature of the GBM, according to Puchalski et al. [[2]] and as described in the text.

**Table S2. Differentially expressed genes between different anatomic features of GBM.** List of genes subjected to multiple *t*-test analyses with a false discovery rate (FDR) approaches using a two-stage step-up method of Benjamini, Krieger, and Yekutieli [[5]]. To create a list of differentially expressed genes between LE *vs*. PZ and CT *vs*. PZ data sets from IvyGAP were used. Differences were considered as statistically significant with FDR<0.01.

**Table S3. List of single gene survival analysis.** List of gene names and statistical information from Kaplan-Meier survival analysis. Tables include information for top 20 genes upregulated in PZ *vs*. LE, top 20 upregulated in LE and CT *vs*. PZ, and of 10-gene oxidative stress signature.

**Table S4. KEGG pathways, GO functional categories and cellular component for genes differentially expressed between studied anatomical features of GBM.** List of top 10 enriched biological terms found in KEGG or GO analysis using 20 most up- and down-regulated genes in comparison LE *vs*. PZ and CT *vs*. PZ or combined 29 genes with prognostic value for patient survival. Functional categories, gene symbols, enrichment FDR, number of genes within the pathway, and an estimated number of genes in the hypothetical pathway are presented in separate columns.

**Table S5. List of genes from different anatomical features of GBM compared with VennDiagram.** Differentially expressed genes between LE, CT, and PZ of GBM were compared using VennDiagram. Genes with the common or opposite direction of changes between studied anatomical features were included in the table.

Supplementary References

1. Verhaak, R.G.; Hoadley, K.A.; Purdom, E.; Wang, V.; Qi, Y.; Wilkerson, M.D.; Miller, C.R.; Ding, L.; Golub, T.; Mesirov, J.P., et al. Integrated genomic analysis identifies clinically relevant subtypes of glioblastoma characterized by abnormalities in PDGFRA, IDH1, EGFR, and NF1. *Cancer Cell* **2010**, *17*, 98-110, doi:10.1016/j.ccr.2009.12.020.

2. Puchalski, R.B.; Shah, N.; Miller, J.; Dalley, R.; Nomura, S.R.; Yoon, J.G.; Smith, K.A.; Lankerovich, M.; Bertagnolli, D.; Bickley, K., et al. An anatomic transcriptional atlas of human glioblastoma. *Science* **2018**, *360*, 660-663, doi:10.1126/science.aaf2666.

3. Celiku, O.; Johnson, S.; Zhao, S.; Camphausen, K.; Shankavaram, U. Visualizing molecular profiles of glioblastoma with GBM-BioDP. *PLoS One* **2014**, *9*, e101239, doi:10.1371/journal.pone.0101239.

4. Szklarczyk, D.; Morris, J.H.; Cook, H.; Kuhn, M.; Wyder, S.; Simonovic, M.; Santos, A.; Doncheva, N.T.; Roth, A.; Bork, P., et al. The STRING database in 2017: quality-controlled protein-protein association networks, made broadly accessible. *Nucleic Acids Res* **2017**, *45*, D362-D368, doi:10.1093/nar/gkw937.

5. Benjamini, Y., Krieger, A. M. & Yekutieli, D. Adaptive linear step-up procedures that control the false discovery rate. *Biometrika* **2006**, *93*, 491–507.

| 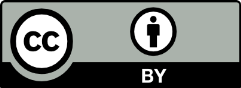 | © 2020 by the authors. Submitted for possible open access publication under the terms and conditions of the Creative Commons Attribution (CC BY) license (http://creativecommons.org/licenses/by/4.0/). |
| --- | --- |
